# Recurrence-free survival is prolonged in high-grade serous ovarian cancer with high PD-L1 expression

**DOI:** 10.1186/s12885-026-16406-3

**Published:** 2026-07-08

**Authors:** Alexandros Lalos, Marina Lovrinovic, Katharina Andersen, Alberto Posabella, Uwe Güth, Sylvia Stadlmann, Tarik Delko, Raoul A. Droeser, Gad Singer

**Affiliations:** 1 Clarunis - University Digestive Health Care Center, St. Clara Hospital, University Hospital of Basel, Spitalstrasse 21, Basel, 4031 Switzerland; 2 Faculty of Medicine, University of Basel, Petersgraben 4, Basel, 4031 Switzerland; 3 Institute of Pathology, Kantonsspital Baden AG, Im Ergel 1, Baden, 5404 Switzerland; 4 Chirurgie Zentrum St. Anna, Luetzelmattstrasse 1, Lucerne, 6006 Switzerland; 5 University Center for Gastrointestinal and Liver Diseases (Clarunis), University of Basel, Spitalstrasse 21, Basel, 4031 Switzerland

**Keywords:** High-grade serous ovarian cancer, PD-1, PD-L1, Tissue microarray, Immunohistochemistry, Prognosis, Biomarker, Recurrence, Survival, Chemosensitivity

## Abstract

**Background:**

High-grade serous ovarian carcinoma (HGSOC) is the most common and clinically aggressive epithelial ovarian cancer subtype and is frequently characterized by relapse despite standard treatment. The FIGO staging system guides treatment decisions and provides prognostic information, but patients within the same stage may display markedly different treatment responses and survival outcomes. Increasing evidence indicates that the tumor microenvironment contributes to recurrence and therapeutic responsiveness. This study focused on the PD-1/PD-L1 immune-checkpoint pathway and evaluated PD-1/PD-L1-positive immune-cell density as a potential prognostic biomarker in HGSOC.

**Methods:**

This retrospective study included 47 patients with HGSOC and matched recurrent tumor samples. Immunohistochemistry was performed on tissue microarrays constructed from paired primary and recurrent formalin-fixed paraffin-embedded samples. PD-1 and PD-L1 immune-cell densities were quantified on evaluable TMA cores, dichotomized into low- and high-density groups, and correlated with recurrence-free survival (RFS), overall survival (OS), chemosensitivity, and clinicopathological parameters. Kaplan-Meier and Cox regression analyses were used to evaluate survival associations.

**Results:**

Patients with high primary PD-L1 immune-cell density had longer RFS than those with low PD-L1 density (log-rank *p* = 0.032). In contrast, no significant difference in RFS was observed between patients with high versus low PD-1 immune-cell density (*p* = 0.18). Neither PD-L1 nor PD-1 density showed a statistically significant association with OS in this cohort.

**Conclusion:**

In this retrospective TMA-based HGSOC cohort, high PD-L1 immune-cell density in the primary tumor was associated with prolonged RFS. These findings should be considered hypothesis-generating and warrant validation in larger, prospectively collected HGSOC cohorts before clinical use.

**Supplementary Information:**

The online version contains supplementary material available at https://doi.org/10.1186/s12885-026-16406-3.

## Introduction

Ovarian cancer (OC) remains the most lethal gynecologic malignancy, largely because most patients are diagnosed with advanced-stage disease and ultimately relapse despite standard treatment [1, 2]. High-grade serous ovarian carcinoma (HGSOC) is the most common and clinically most aggressive epithelial OC subtype [3–5]. Although histology and stage guide management, patients with apparently similar HGSOC frequently experience markedly different recurrence patterns, treatment responses, and survival outcomes. Standard management of HGSOC includes cytoreductive surgery followed by platinum-based chemotherapy, but recurrence is common and platinum resistance emerges in a substantial subset of patients [5, 6]. Clinical staging remains essential for treatment planning; however, it does not fully capture inter-patient heterogeneity in recurrence risk and treatment benefit [5–8].

In recent years, growing attention has turned to the tumor microenvironment (TME) as a key contributor to cancer progression and treatment response. The TME comprises a complex network of stromal, immune, inflammatory, and tumor-associated factors that influence tumor growth, metastatic potential, and immune escape [9–11]. Immune checkpoint (IC) pathways are central to immune homeostasis, preventing overactivation and maintaining tolerance during immune responses [12, 13]. In cancer, however, these pathways may be co-opted by tumor cells and tumor-associated immune cells to dampen antitumor immunity and facilitate immune evasion [12, 13]. Previous work from this research group and others has linked immune-related biomarkers, including OX40, IL-17, CD16+, and CD66b-positive tumor-infiltrating immune cells, with chemotherapy responsiveness and clinical outcome in ovarian carcinoma [14–17].

The present study focused on programmed cell death protein 1 (PD-1) and its ligand, programmed death-ligand 1 (PD-L1). PD-1 is primarily expressed on activated immune cells, including tumor-infiltrating lymphocytes, whereas PD-L1 may be expressed by antigen-presenting cells, tumor-associated immune cells, and tumor cells [12, 13, 18]. The PD-1/PD-L1 interaction suppresses T-cell activation and can contribute to immune evasion, but its prognostic significance varies across tumor types and cellular compartments [19–22]. In ovarian carcinoma, prior studies have reported increased PD-1/PD-L1 expression in high-grade tumors, but the clinical relevance of this pathway remains incompletely defined [23, 24]. With these considerations in mind, PD-1- and PD-L1-positive tumor-infiltrating immune cells were evaluated in paired primary and recurrent HGSOC, with emphasis on associations with recurrence-free interval, chemosensitivity, and overall survival.

## Materials and methods

### Patients’ and tumor characteristics

A retrospective series of 47 women with HGSOC and matched recurrences was assembled from four Swiss pathology centers (Institute of Pathology of the University Hospital of Basel and the Cantonal Hospitals of Liestal, St. Gallen, and Baden). Cases were diagnosed and treated between 1985 and 2003, consistent with the established archival HGSOC TMA cohort from these institutions. The cohort was not strictly consecutive; cases were included when matched primary and recurrent FFPE tumor material and corresponding clinicopathological follow-up data were available. Eligibility required primary debulking surgery followed by at least three cycles of platinum-based chemotherapy. All patients subsequently relapsed and were categorized as chemorefractory/resistant (recurrence ≤ 6 months after adjuvant therapy) or chemosensitive (recurrence > 6 months after adjuvant therapy). Clinicopathological variables and survival outcomes were abstracted from institutional records.

### Tissue microarray (TMA) construction

Representative tumor areas were identified on corresponding H&E-stained full-face sections before TMA construction. From each available FFPE donor block, one representative 1-mm core was transferred into recipient blocks to create TMAs following established protocols [15, 25]. Each core was taken from viable tumor regions with more than 50% tumor cells, and matched recurrent samples were processed analogously when suitable tissue was available. Tissue cylinders with a diameter of 1 mm were punched from morphologically typical sections of each donor block and placed into a recipient paraffin block (30 × 25 mm). Methodological details on TMA assembly and layout followed prior descriptions [15, 25].

### Immunohistochemistry

**Fig. 1:**
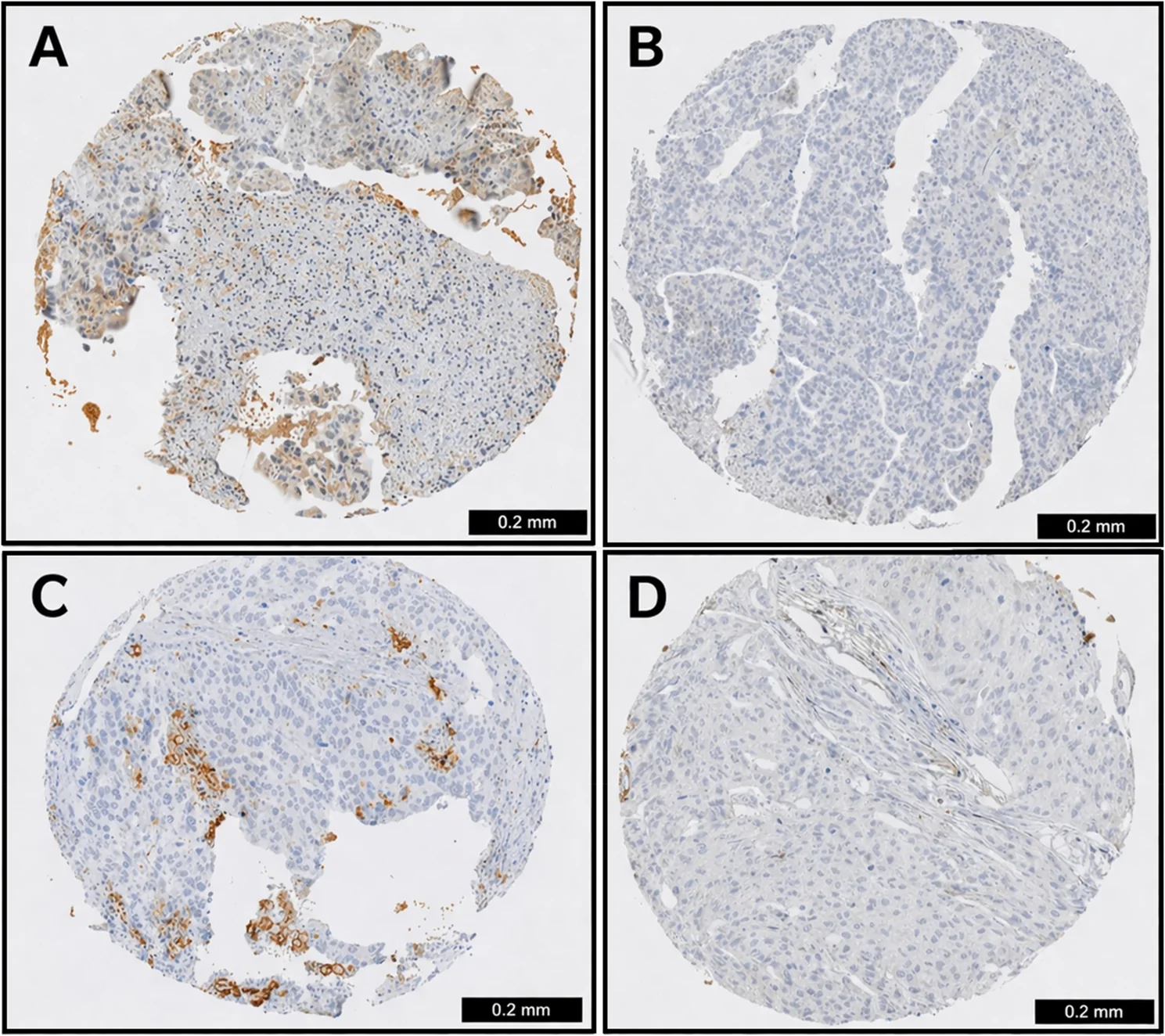
Representative immunohistochemical images of TMA cores showing high (**A**) and low (**B**) PD-L1-positive immune-cell density and high (**C**) and low (**D**) PD-1-positive immune-cell density (20× magnification)

Immunohistochemical (IHC) staining was performed on 4 μm sections from FFPE recipient TMA blocks using anti-PD-L1 and anti-PD-1 antibodies on a Benchmark platform (Ventana Medical Systems) with iVIEW-DAB chromogen, as described previously by this research group [14–17]. Sections were pre-treated with CC1 for 16 min and incubated with the primary antibodies at a dilution of 1:400 for 20 min. The antibody details were as follows: anti-PD-L1 (clone SP263; Ventana Medical Systems; catalog no. 790–4905) and anti-PD-1 (Ventana Medical Systems; catalog no. 760–4895). Slides were digitized using an Olympus VS200 slide scanner. Areas with necrosis, staining artifacts, or ≤ 25% preserved tumor tissue were excluded. A 20× high-power field (HPF) was used as the reference field for density-based scoring, and the dichotomization threshold (high vs. low) was derived in this cohort by regression-tree analysis (cut-off: 16 positive immune cells per 20× HPF-equivalent field), following established approaches for selecting IHC cut-offs [26]. Figure 1 shows representative high versus low staining patterns.

### Evaluation of immunohistochemistry

Two pathologists, blinded to clinical data, quantified PD-1- and PD-L1-positive tumor-infiltrating immune cells on each evaluable TMA core and recorded counts as cell density per 20× HPF-equivalent area. Because a 1-mm TMA core approximates the area of a single 20× field, the entire evaluable core was assessed rather than selecting a single “best” HPF. When more than one evaluable core was available for a tumor, the mean immune-cell count across evaluable cores was used for analysis. Discordant evaluations between the two pathologists were resolved by joint review and consensus. Scoring focused on immune-cell staining rather than tumor-cell TPS/CPS, in line with HGSOC studies showing that PD-L1 is frequently observed in immune/stromal compartments and that immune-cell and tumor-cell PD-L1 can carry different prognostic associations [27–29]. Tumor-cell scoring would require whole-slide assessment and was not part of the prespecified analysis.

### Statistical analysis

Statistical analyses were performed using STATA version 13 (StataCorp, College Station, TX, USA) and R version 4.0.2 (https://www.r-project.org, accessed on 24 May 2021). Recurrence-free survival (RFS) was defined as the time from primary surgery to documented recurrence, and overall survival (OS) was defined as the time from primary surgery to death or last follow-up. Cut-off values used to classify HGSOC with low or high immune-cell infiltration were derived from previous TMA-based ovarian carcinoma biomarker studies15-18 or generated by regression-tree analysis, following established recommendations for biomarker cut-off selection27. Kruskal-Wallis, Chi-square, and Fisher’s exact tests were used to explore associations between clinicopathological features and biomarker-defined groups, and Kaplan-Meier survival curves were compared using the log-rank test. For the Cox regression analysis, PD-L1 density was modeled as a continuous variable per one additional PD-L1-positive immune cell per 20x HPF-equivalent area, whereas the 16 cells/HPF cut-off was used for dichotomized Kaplan-Meier stratification. P-values were adjusted for multiple comparisons according to Benjamini and Hochberg. Univariable and multivariable Cox proportional-hazards regression models were used to evaluate RFS; the multivariable model was adjusted for age, FIGO stage, residual disease after cytoreductive surgery, number of chemotherapy cycles, and PD-L1 density. P-values < 0.05 were considered statistically significant.

## Results

### Patient and tumor characteristics

The clinicopathological characteristics of the patients included in the HGSOC cohort are provided in Table 1. The median age was 58 years (range, 34–77 years). Regarding FIGO classification, one patient (2.1%) had FIGO stage II disease, 38 patients (80.9%) had FIGO stage III disease, and 8 patients (17.0%) had FIGO stage IV disease. All patients underwent initial debulking surgery; 16 patients (34.0%) had no macroscopic residual disease, 17 patients (36.2%) had residual disease ≤ 2 cm, and 13 patients (27.7%) had residual disease > 2 cm. After surgery, all patients received at least three cycles of chemotherapy, and 39 patients (83.0%) received six or more cycles. Chemoresistant tumors were defined as those recurring within six months after completion of adjuvant treatment, and 33 patients (70.2%) were classified as chemosensitive. The 6-month RFS rate was 53.0% (95% CI, 38.0%-66.0%), and the 3-year OS rate was 47.0% (95% CI, 29.0%-63.0%).

**Table 1:** Patients’ characteristics of the overall HGSOC cohort (*N* = 47)

	*N* = 47*, (%)
Age (median, range)	58 (34 - 77)
FIGO stage:	
II	1 (2.1)
IIIA	1 (2.1)
IIIB	5 (10.6)
IIIC	32 (68.2)
IV	8 (17.0)
Residual disease:	
None	16 (34.0)
≤ 2 cm	17 (36.2)
> 2 cm	13 (27.7)
Number of Chemotherapy cycles:	
< 6	8 (17.0)
6 or more	39 (83.0)
Response to Chemotherapy	
CS	33 (70.2)
CR	14 (29.8)
RFS in months	10.1 (9.89 - 10.30)
OS in months	41.4 (40.77 - 42.03)
6-month RFS, % (95% CI)	53 (95% CI, 38-66)
3-year OS, % (95% CI)	47 (95% CI, 29%-63)

*CI* confidence interval, *CR* chemoresistant, *CS* chemosensitive, *OS* overall survival, *RFS* recurrence-free survival

*Missing clinicopathological information was assumed to be missing at random

### PD-1 expression in primary and recurrent HGSOC and its association with clinicopathological features

First, PD-1 expression was assessed in the TMA to investigate potential correlations with clinicopathological features. No significant associations were identified between PD-1 density and clinical outcomes, including OS, RFS, and chemosensitivity, in primary HGSOC samples (Table 2) or matched recurrent samples (Table S1). Other clinicopathological features, including age, FIGO stage, residual disease, number of chemotherapy cycles, and response to chemotherapy, were similarly distributed across the PD-1-low and PD-1-high groups. At the cohort level, PD-1-high and PD-1-low proportions were comparable between primary and recurrent samples, but a paired analysis of within-patient changes would require complete evaluable matched cores for all patients and was not performed in the current study.

**Table 2:** Patients’ characteristics according to dichotomized distribution of PD-1 in primary HGSOC samples (*N* = 44*)

	PD-1 low *N* = 22 (%)	PD-1 high *N* = 22 (%)	*p*-value
Age (median, range)	58 (39 - 77)	58 (34 - 72)	0.778
FIGO stage:			0.409
II	1 (4.5%)	0 (0.0%)	
IIIA	0 (0.0%)	0 (0.0%)	
IIIB	4 (18.2%)	1 (4.5%)	
IIIC	13 (59.1%)	17 (77.3%)	
IV	4 (18.2%)	4 (18.2%)	
Residual disease:			0.741
None	8 (36.4%)	5 (22.7%)	
≤ 2 cm	8 (36.4%)	9 (40.1%)	
> 2 cm	6 (27.3%)	7 (31.8%)	
Number of CT cycles:			0.068
< 6	1 (4.5%)	5 (22.7%)	
6 or more	21 (95.6%)	16 (72.7%)	
Chemotherapy:			0.195
CS	13 (59.1%)	17 (77.3%)	
CR	9 (40.9%)	5 (22.7%)	
6-month RFS, % (95% CI)	50.0% (95% CI, 28.0%-68.0%)	65.0% (95% CI, 42.0%-81.0%)	0.176
3-year OS, % (95% CI)	53.0% (95% CI, 26.0%-74.0%)	40.0% (95% CI, 17.0%-63.0%)	0.983

Variables are indicated as absolute numbers, percentages, medians, or ranges. Age, OS, and RFS were evaluated using the Kruskal-Wallis test. FIGO stage, residual disease, number of chemotherapy cycles, and chemoresistance were analyzed using the Chi-square or Fisher’s exact test

*CI* confidence interval, *CR* chemoresistant, *CS* chemosensitive, *CT* chemotherapy, *OS* overall survival, *RFS* recurrence-free survival

*Percentages may not add to 100% due to missing values of defined variables; missing clinicopathological information was assumed to be missing at random

### PD-L1 expression in primary and recurrent HGSOC and its association with clinicopathological features

Second, PD-L1 expression was evaluated in the TMA and correlated with clinicopathological factors. Patients with high PD-L1 immune-cell density in primary HGSOC samples had longer RFS than patients with low PD-L1 density (log-rank *p* = 0.032). This p-value reflects the log-rank comparison of the full Kaplan-Meier RFS curves rather than a comparison of the 6-month RFS percentages alone. No significant difference in OS was observed between the PD-L1-low and PD-L1-high groups. These findings are presented in detail in Table 3. Other clinicopathological characteristics, including age, FIGO stage, residual disease, number of chemotherapy cycles, and response to treatment, showed similar distributions between the two PD-L1 expression groups, mirroring the pattern observed for PD-1. Conversely, in matched recurrent HGSOC samples, no correlation between PD-L1 expression and clinical outcomes was identified (Table S2). Overall, the proportion of PD-L1-high cases was similar in primary and recurrent samples at the cohort level; however, formal paired within-patient change analyses were limited by missing evaluable cores in some matched cases and were not part of the prespecified statistical plan.

**Table 3:** Patients’ characteristics according to dichotomized distribution of PD-L1 in primary HGSOC samples (*N* = 45*)

	PD-L1 low *N* = 26 (%)	PD-L1 high *N* = 19 (%)	*p*-value
Age (median, range)	58.5 (39–77)	58 (34–69)	0.527
FIGO stage:			0.932
II	1 (3.8%)	0 (0.0%)	
IIIA	0 (0.0%)	0 (0.0%)	
IIIB	2 (7.7%)	3 (15.8%)	
IIIC	19 (73.1%)	13 (68.4%)	
IV	4 (15.4%)	3 (15.8%)	
Residual disease:			1.000
None	9 (34.6%)	6 (31.6%)	
≤ 2 cm	10 (38.5%)	7 (36.8%)	
> 2 cm	7 (26.9%)	5 (26.3%)	
Number of CT cycles:			0.356
< 6	2 (7.7%)	3 (15.8%)	
6 or more	24 (92.3%)	15 (78.9%)	
Response to Chemotherapy:			0.734
CS	19 (73.1%)	13 (68.4%)	
CR	7 (26.9%)	6 (31.6%)	
6-month RFS, % (95% CI)	58.0% (95% CI, 37.0%-74.0%)	63.0% (95% CI, 38.0%-81.0%)	0.032
3-year OS, % (95% CI)	50.0% (95% CI, 26.0%-70.0%)	39.0% (95% CI, 14.0%-63.0%)	0.974

Variables are indicated as absolute numbers, percentages, medians, or ranges. Age, OS, and RFS were evaluated using the Kruskal-Wallis test. FIGO stage, residual disease, number of chemotherapy cycles, and chemoresistance were analyzed using the Chi-square or Fisher’s exact test

*CI* confidence interval, *CR* chemoresistant, *CS* chemosensitive, *CT* chemotherapy, *OS* overall survival, *RFS* recurrence-free survival

*Percentages may not add to 100% due to missing values of defined variables; missing clinicopathological information was assumed to be missing at random

### Kaplan-Meier and multivariable Cox regression analyses of RFS in primary HGSOC

**Fig. 2:**
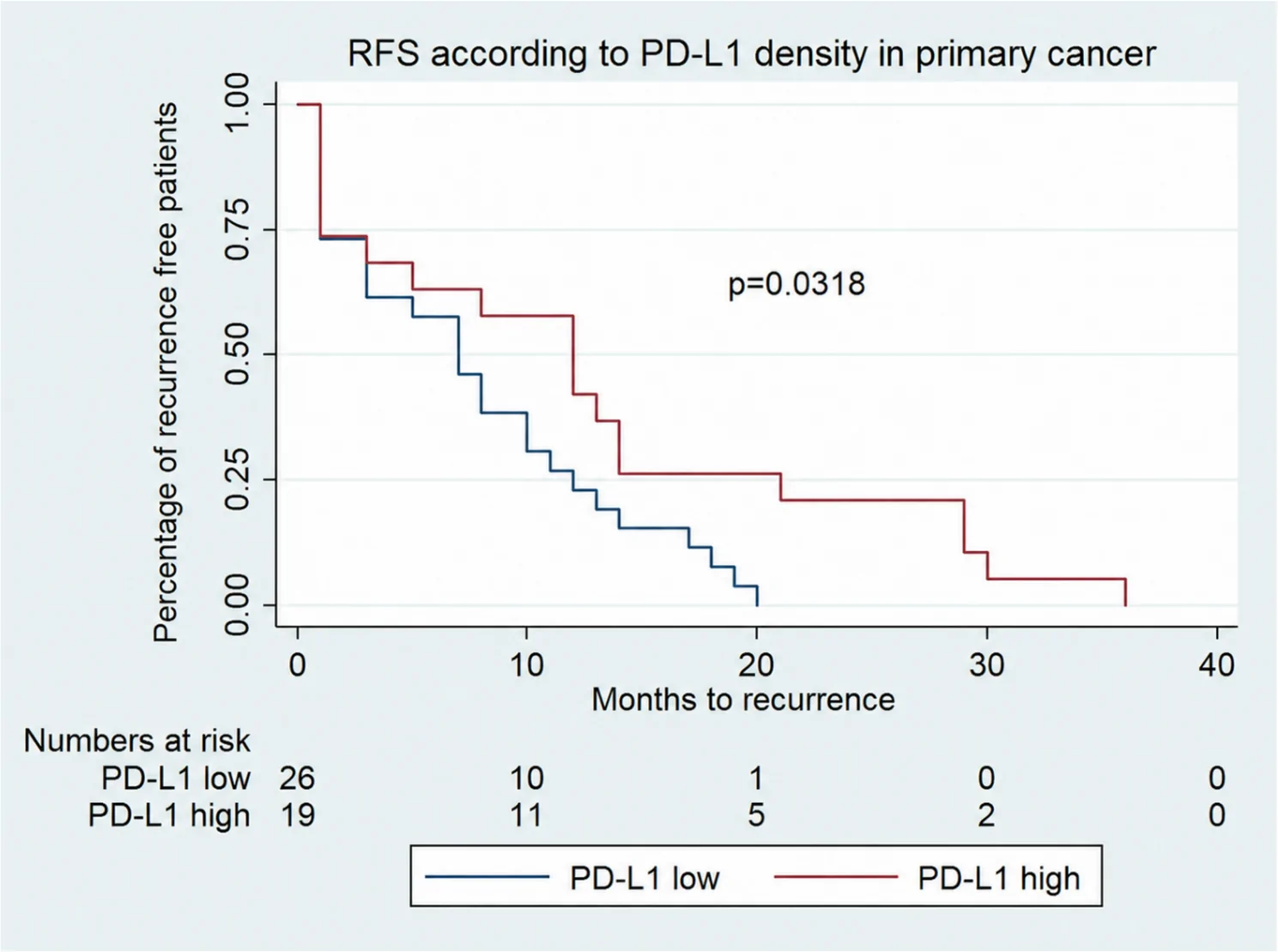
Kaplan-Meier curves for RFS in primary HGSOC, stratified by PD-L1 immune-cell density (high vs. low). Kaplan-Meier curves comparing RFS between primary HGSOC cases with low and high PD-L1 immune-cell density. The blue line indicates tumors with low PD-L1 density, and the red line indicates tumors with high PD-L1 density. The x-axis shows months to recurrence, and the y-axis shows recurrence-free survival probability. The p-value was calculated using the log-rank test

The recurrence-free interval was longer in primary HGSOC cases with high PD-L1 immune-cell density than in tumors with low PD-L1 density, as shown by Kaplan-Meier analysis (Fig. 2). In matched recurrent HGSOC samples, Kaplan-Meier analysis did not identify a significant association between PD-L1 density and survival outcomes. To further examine associations between clinicopathological features and PD-L1 density, multivariable Cox proportional-hazards models were fitted including age, residual disease, FIGO stage, number of chemotherapy cycles, and PD-L1 density. PD-L1 density was modeled continuously in the Cox model, rather than as the dichotomized > 16 versus < = 16 cells/HPF variable used for Kaplan-Meier stratification. Higher continuous PD-L1 density was associated with a lower hazard of recurrence (adjusted HR 0.985 per additional PD-L1-positive immune cell/HPF, 95% CI 0.973–0.997, *p* = 0.015; residual disease > 2 cm: adjusted HR 4.14, 95% CI 1.62–10.56, *p* = 0.003; Table 4). Because this HR is expressed per one-cell increment, the clinical effect of a single additional cell is small; the result should be interpreted as indicating a gradual inverse association between increasing PD-L1 immune-cell density and recurrence hazard, rather than as an independent clinical threshold.

**Table 4:** Multivariable Cox regression analysis of RFS in primary HGSOC

	Multivariable		
	HR	95% CI	*p*-value
Age	1.01	0.97–1.04	0.782
PD-L1 density (continuous; per 1 positive immune cell/HPF)	0.985	0.973–0.997	0.015
Residual disease ≤ 2 cm	1.31	0.59–2.89	0.494
Residual disease > 2 cm	4.14	1.62–10.56	0.003
Number of chemotherapy cycles	2.26	0.79–6.39	0.124
FIGO IIIB	0.83	0.09–7.83	0.873
FIGO IIIC	1.25	0.16–10.08	0.833
FIGO IV	1.65	0.18–14.84	0.656

Multivariable analyses showing hazard ratios and p-values for primary HGSOC samples, adjusted for PD-L1 density, age, residual disease after cytoreductive surgery, number of chemotherapy cycles, and FIGO stage. PD-L1 density was modeled as a continuous variable; an HR below 1.0 indicates a lower recurrence hazard with increasing PD-L1-positive immune-cell density. The dichotomized cut-off of 16 cells/HPF was used for Kaplan-Meier stratification and descriptive group comparisons

*CI* confidence interval, *FIGO* Fédération Internationale de Gynécologie et d’Obstétrique, *HPF* high-power field, *HR* hazard ratio, *RFS* recurrence-free survival

Figure 2. Kaplan-Meier curves comparing RFS between primary HGSOC cases with low and high PD-L1 immune-cell density. The blue line indicates tumors with low PD-L1 density, and the red line indicates tumors with high PD-L1 density. The x-axis shows months to recurrence, and the y-axis shows recurrence-free survival probability. The *p*-value was calculated using the log-rank test.

## Discussion

Despite advances in surgical techniques and the routine use of adjuvant chemotherapy, the prognosis of HGSOC remains poor. One major contributor to this unfavorable outcome is the high recurrence rate, with many patients experiencing relapse after initial standard treatment [5, 30–34]. In recurrent HGSOC, platinum-based therapy, used alone or in combination and with or without bevacizumab, remains central to treatment. Prognosis at relapse is strongly influenced by platinum sensitivity, and patients who relapse during first-line treatment or shortly thereafter represent a biologically heterogeneous population [30–34]. Repeated systemic treatment is often associated with considerable toxicity, making biomarkers that reflect tumor biology, treatment responsiveness, or prolonged RFS clinically relevant.

Although major progress has been made in understanding the role of the TME in ovarian carcinoma, important questions remain unresolved. Immune checkpoints are essential for maintaining self-tolerance, but tumors may exploit these pathways to evade immune surveillance [12, 13, 18–24]. PD-1 is expressed on activated immune cells, and its ligand PD-L1 has been linked to both immune regulation and tumor progression. In HGSOC, however, the prognostic significance of PD-L1 remains inconsistent, with studies reporting favorable or unfavorable associations depending on the cellular compartment examined and the scoring methodology applied [19, 20, 23, 24, 27–29]. Martín de la Fuente et al. reported that PD-L1 expression in advanced HGSOC was observed mainly in macrophages and that higher PD-L1 and PD-1 expression, together with increased tumor-infiltrating lymphocytes, was associated with improved survival [28]. Similarly, Kim et al. found that PD-L1 expression on stromal tumor-infiltrating lymphocytes was positively associated with survival in ovarian serous carcinoma [29].

In the present cohort, high PD-L1 expression in the tumor microenvironment was significantly associated with prolonged RFS, whereas no significant association with OS was identified. PD-1 expression was not significantly associated with RFS or OS. Because all patients included in the primary-tumor cohort subsequently developed recurrence, the purpose of the primary analysis was not to compare patients with versus without recurrence, but rather to determine whether PD-1/PD-L1 expression in the primary tumor stratified the length of the recurrence-free interval. Within that framework, high PD-L1 immune-cell density may mark tumors with more favorable biology and/or greater chemosensitivity [15–17, 28, 29, 35]. One possible interpretation is that PD-L1 positivity in the immune/stromal compartment reflects an immune-inflamed TME rather than a purely tumor-intrinsic immune-evasion phenotype. In HGSOC, favorable prognosis has repeatedly been linked to abundant CD8 + tumor-infiltrating lymphocytes [27, 28, 35]. Accordingly, PD-L1-positive immune infiltrates may, at least in a subset of tumors, accompany an ongoing antitumor immune response rather than simply indicate immune escape [27–29, 35].

At the same time, the published literature remains heterogeneous. Differences in histologic subtype, tissue compartment, antibody clone, scoring method, and whether PD-L1 is assessed in immune cells or tumor cells likely contribute to variability across studies [20, 23, 24, 27–29]. This methodological heterogeneity may also help explain why recurrent-tumor analyses in this cohort did not show significant associations with survival endpoints. One limitation of the study is its retrospective design, which may introduce selection bias and limit generalizability despite inclusion of patients from four centers. The relatively small sample size further constrains statistical power and precludes robust subgroup analyses. In addition, TMA-based assessment samples only a fraction of the tumor and may not fully capture intratumoral heterogeneity.

PD-1- and PD-L1-positive immune cells were quantified morphologically on single-stain sections without multiplex immune phenotyping; therefore, some misclassification of cell type cannot be fully excluded and should be addressed in future work using double staining or multiplex approaches. There is also no universally accepted ovarian-specific PD-L1 scoring standard. Companion diagnostic algorithms such as TPS and CPS were developed for other tumor types and require a tumor-cell denominator on whole-slide sections, whereas the prespecified approach used here quantified PD-1/PD-L1-positive tumor-infiltrating immune cells within a fixed 20× HPF-equivalent area on 1-mm TMA cores and dichotomized cases using a data-driven cut-off of 16 cells/HPF. From a practical perspective, these data do not suggest that PD-L1 alone should guide treatment decisions. Rather, if confirmed in larger prospective cohorts, PD-L1 immune-cell density could complement established clinicopathologic factors such as stage, residual disease, and treatment response. Combining PD-L1 with markers of effector immunity, particularly CD8 + tumor-infiltrating lymphocytes, and with other immune subsets previously linked to chemosensitivity in HGSOC may ultimately provide a more informative biologic risk-stratification framework than any single marker alone.

## Conclusion

High PD-L1 immune-cell density in primary HGSOC was associated with longer RFS in this retrospective cohort. The finding supports the hypothesis that PD-L1-positive immune infiltrates may identify a subset of tumors with a more immune-inflamed microenvironment and potentially more favorable recurrence biology. Because the study is retrospective, relatively small, and based on TMA-derived immune-cell scoring, the results should be interpreted cautiously. Validation in larger prospective HGSOC cohorts with standardized scoring and complementary immune phenotyping is required before clinical implementation.

## Supplementary Information

Supplementary Material 1.

## Data Availability

Raw data can be obtained by contacting the corresponding author.

## Abbreviations

CI: Confidence interval
CPS: Combined Positive Score
CR: Chemoresistant
CS: Chemosensitive
EKNZ: Ethics Committee of Northwestern Switzerland
FFPE: Formalin-Fixed Paraffin-Embedded
FIGO: Fédération Internationale de Gynécologie et d’Obstétrique / International Federation of Gynecology and Obstetrics
HPF: High-Power Field
HGSOC: High-Grade Serous Ovarian Carcinoma
IC: Immune Checkpoint
IHC: Immunohistochemistry
NK: Natural Killer
OC: Ovarian Cancer
OS: Overall Survival
PD-1: Programmed Cell Death 1
PD-L1: Programmed Death-Ligand 1
RFS: Recurrence-Free Survival
STATA: Software for Statistics and Data Science
TICs: Tumor-Infiltrating Cells
TILs: Tumor-Infiltrating Lymphocytes
TMA: Tissue Microarray
TME: Tumor Microenvironment
TNM: Tumor, Node, Metastasis
TPS: Tumor Proportion Score
