# Supplementary material for: Recurrence-free survival is prolonged in high-grade serous ovarian cancer with high PD-L1 expression
Source: BMC Cancer. 2026 Jul 8;26:1151. doi: 10.1186/s12885-026-16406-3 (PMC13628871; doi:10.1186/s12885-026-16406-3)
Supplement: Supplementary file 1 [file 12885_2026_16406_MOESM1_ESM.docx]

**9. Supplementary tables**

**Table S1. Patients’ characteristics according to dichotomized distribution of PD-1 in recurrent HGSOC samples (N = 39*)**

|  | PD-1 low  N = 20 (%) | PD-1 high  N = 19 (%) | p-value |
| --- | --- | --- | --- |
| Age (median, range) | 57.5 (34 - 76) | 58 (41 - 73) | 0.789 |
| FIGO stage:  II  IIIA  IIIB  IIIC  IV | 0 (0.0%) 1 (5.0%) 0 (0.0%) 16 (80.0%) 3 (15.0%) | 1 (5.3%) 0 (0.0%) 5 (26.3%) 9 (47.4%) 4 (21.1%) | 0.028 |
| Residual disease:  None  ≤ 2 cm  > 2 cm | 9 (45.0%) 5 (25.0%) 5 (25.0%) | 4 (21.1%) 10 (52.6%) 5 (26.3%) | 0.150 |
| Number of CT cycles:  < 6  6 or more | 4 (20.0%) 15 (75.0%) | 1 (5.3%) 18 (94.7%) | 0.150 |
| Response to Chemotherapy:  CS  CR | 12 (60.0%) 8 (40.0%) | 15 (78.9%) 4 (21.1%) | 0.200 |
| 6-month RFS, % (95% CI) | 50.0% (95% CI, 27.0%-69.0%) | 68.0% (95% CI, 43.0%-84.0%) | 0.822 |
| 3-year OS, % (95% CI) | 50.0% (95% CI, 24.0%-71.0%) | 54.0% (95% CI, 14.0%-66.0%) | 0.98 |

*Percentages may not add to 100% due to missing values of defined variables; missing clinicopathological information was assumed to be missing at random. Variables are indicated as absolute numbers, percentages, medians, or ranges. Age, OS, and RFS were evaluated using the Kruskal-Wallis test. FIGO stage, residual disease, number of chemotherapy cycles, and chemoresistance were analyzed using the Chi-square or Fisher’s exact test. CI: confidence interval; CR: chemoresistant; CS: chemosensitive; CT: chemotherapy; OS: overall survival; RFS: recurrence-free survival.

**Table S2. Patients' characteristics according to dichotomized distribution of PD-L1 in recurrent HGSOC samples (N = 43*)**

|  | PD-L1 low  N = 23 (%) | PD-L1 high  N = 20 (%) | p-value |
| --- | --- | --- | --- |
| Age (median, range) | 58 (39 - 77) | 55.5 (43 - 69) | 0.558 |
| FIGO stage:  II  IIIA  IIIB  IIIC  IV | 1 (4.3%) 0 (0.0%) 1 (4.3%) 16 (69.6%) 5 (21.7%) | 0 (0.0%) 1 (5.0%) 4 (20.0%) 12 (60.0%) 3 (15.0%) | 0.295 |
| Residual disease:  None  ≤ 2 cm  > 2 cm | 8 (34.8%)  9 (39.1%)  5 (21.7%) | 7 (35.0%)  7 (35.0%)  6 (30.0%) | 0.926 |
| Number of CT cycles:  < 6  6 or more | 2 (8.7%)  20 (86.9%) | 4 (20.0%)  16 (80.0%) | 0.313 |
| Response to Chemotherapy:  CS  CR | 17 (73.9%)  6 (26.1%) | 14 (70.0%)  6 (30.0%) | 0.775 |
| 6-month RFS, % (95% CI) | 65.0% (95% CI, 42.0%-81.0%) | 55.0% (95% CI, 31.0%-73.0%) | 0.643 |
| 3-year OS, % (95% CI) | 44.0% (95% CI, 21.0%-65.0%) | 45.0% (95% CI, 17.0%-71.0%) | > 0.1 |

*Percentages may not add to 100% due to missing values of defined variables; missing clinicopathological information was assumed to be missing at random. Variables are indicated as absolute numbers, percentages, medians, or ranges. Age, OS, and RFS were evaluated using the Kruskal-Wallis test. FIGO stage, residual disease, number of chemotherapy cycles, and chemoresistance were analyzed using the Chi-square or Fisher’s exact test. CI: confidence interval; CR: chemoresistant; CS: chemosensitive; CT: chemotherapy; OS: overall survival; RFS: recurrence-free survival.
